# Experimental assessment of 3D-printed traps and chemical attractants for the collection of wild *Drosophila melanogaster*

**DOI:** 10.1080/19336934.2025.2502184

**Published:** 2025-05-14

**Authors:** Alexandra H. Keene-Snickers, Tillie J. Dunham, Mark D. Stenglein

**Affiliations:** a Center for Vector-Borne and Infectious Diseases, Department of Microbiology, Immunology, and Pathology, College of Veterinary Medicine and Biomedical Sciences, Colorado State University, Fort Collins, CO, USA; b Quantitative Cell and Molecular Biology Graduate Program, Colorado State University, Fot Collins, CO, USA

**Keywords:** *Drosophila melanogaster*, wild *Drosophila*, microbiota, fly collection, citizen science, field application, marula fruit, crowdsourced sample collection

## Abstract

*Drosophila melanogaster*, the common fruit fly, has been instrumental to our understanding of evolution, genetics and disease. There are benefits to studying these flies in the wild, including assessment of their naturally occurring microbiota. To facilitate efforts to catch wild *D. melanogaster*, we designed two fly traps and evaluated several candidate attractants. The first trap utilized a stable food substrate that can be used to catch live flies to establish new lab colonies. The second trap was designed to be reusable and easy to ship to enable the collection of flies over time from diverse locations. We evaluated several chemical attractants derived from banana and from marula fruit, which is the proposed ancestral food host of *D. melanogaster*. We found that wild flies were preferentially attracted to banana-based odorants over marula-derived ones. Overall, these traps and attractants represent an inexpensive and simple option for the collection of wild *D. melanogaster* and related species for sampling or colony establishment.

## Introduction

Research involving *Drosophila melanogaster* has led to major advances in our understanding of genetics, molecular and cellular biology and disease [1]. Although laboratory fly lines are useful, neither their genetics nor the laboratory environment necessarily recapitulates the situation in the wild. Studies of *Drosophila* evolution, microbiome and virus infection have benefited from the use of wild-caught flies [2–5].

The study of the wild fly microbiota reflects an increasing general interest in the natural microbiota of model organisms. For instance, viruses isolated from wild-caught *Caenorhabditis* nematodes have enabled studies of small interfering RNA responses to natural virus infection [6]. Wilding mice, created by implanting laboratory mouse embryos into wild mothers, exhibit a more natural immune response [7,8]. Similarly, wild *D. melanogaster* harbour diverse microbes not necessarily present in laboratory strains. These interact with the host and other microbiota, altering host biology and evolution [2,9–12]. The capture and maintenance of wild derived flies in the lab has been instrumental to understanding the impact of common *D. melanogaster* infecting viruses [2,3,9,13–15].

To capture wild *D. melanogaster*, researchers typically use manual or electronic aspirators, net swipes, or baited traps consisting of fruit (commonly banana) and yeast [16–19]. Other trap types aim to attract and kill pest *Drosophila* species. *Drosophila suzukii*, a major agricultural pest of soft fruits, can be efficiently captured and killed using vinegar-based traps [20–22]. The attractiveness of a trap is important to how successful it will be, particularly in natural environments where there is competition from other food sources.

In this study, we experimentally assessed the stability and attractiveness of different bait substrates, trap designs and banana- and marula-derived attractants. The marula fruit is proposed to have been an important host fruit for ancestral *D. melanogaster* ^*23*^. Populations of *D. melanogaster* in sub-Saharan Africa have polymorphisms in the Or22a-expressing olfactory sensory neurons that increase their sensitivity to ethyl isovalerate, a compound in marula fruit [23]. Other compounds in marula fruit include isoamyl acetate, which is also a main component of synthetic banana flavouring.

Our goal was to design traps that gave us the ability to collect and retain live flies if we wanted and to be easily used by participatory scientists for crowdsourced fly collection. To this end, we designed two traps. The first trap focused on the capture of live flies for the generation of new lab colonies. This trap relied on an environmentally stable cornmeal-based food and the addition of isoamyl acetate to increase attractiveness. Our second trap was designed with ease of use in mind. This trap used banana and yeast, a maximally attractive and readily available bait, and includes a mechanism to keep flies from getting stuck in the bait. These traps could also be reused, making them useful for longitudinal collections.

## Methods and materials

### Fly food

Cornmeal-based food: We used the standard cornmeal food-based recipe from the Bloomington Drosophila Stock Center (https://bdsc.indiana.edu/information/recipes/bloomfood.html). Twenty-five to 50 mL of food was placed in fly bottes (VWR 75813–140). For experiments involving ethyl isovalerate, ethyl isovalerate (Sigma-Aldrich 112283) was added to the final per cent volume concentrations of 0.45%, 0.9%, 1.8%, 3.6% or 7.2%. For experiments involving isoamyl acetate, synthetic banana flavouring (Kroger Brand Imitation Banana, 87–9110) was added to a final concentration of 1.5%.

Potato-based food was prepared as described by Waddle, 1996 (www.anapsid.org/fruitfly.html), except baker’s yeast was substituted for brewer’s yeast [24]. Briefly, 120 g of instant potato was mixed with 12 g yeast. Mould inhibitor was made by adding 1 g of Tegosept (Sigma-Aldrich, H3647) to 1 L of boiling water. The diluted Tegosept was then added to the potato/yeast mixture to rehydrate the potatoes. The volume of mould inhibitor varied from batch to batch based on how much liquid was needed to fully rehydrate the potato/yeast mixture. Twenty-five to 50 mL of food was then placed in fly bottles and sprinkled with baker’s yeast. For attractiveness experiments, 4 g of agar dissolved in 200 mL water was added to increase shelf life.

Banana-based food was prepared as described by Hundt, 1996 (www.anapsid.org/fruitfly.html) [24]. Briefly, four bananas were mashed together with 1/8 cup cane sugar until liquified. Rolled oats were added until the mixture became firm. Twenty-five to 50 mL of food was transferred to fly bottles and sprinkled with baker’s yeast.

### Fly food attractants

All potential attractants except the homemade banana extract were obtained through commercial vendors: isoamyl acetate (Kroger Brand Imitation Banana, 87–9110), ethyl isovalerate (Sigma-Aldrich 112283) and marula oil (Rocky Mountain Oil Company, All Organic Marula Oil). Homemade banana extract was made by manually mashing bananas until mostly liquid. The pulp and liquid were then separated using a mesh strainer. The liquid was retained and stored in a −20°C freezer until use.

### Outdoor experiments

For attractiveness experiments, wild-derived flies from our lab’s FoCo17 colony were used [25]. Microcosms were constructed using bug dorms (dimensions: W32.5 × D32.5 × H77.0 cm; similar to: bugdorm, BD4F3074). Microcosms were protected from rain by a tarp. Flies released into the microcosms were given 24–48 h to select one of the foods, either plain or supplemented with one of the marula fruit or banana additives. To trap the flies, we used food bottles capped with 3D printed trap lids. These lids allowed flies to easily enter the bottle but not as easily get out. Bottles were collected and frozen before the number of flies in each bottle was counted.

### Indoor experiments

Population cages in our insectary were used for these experiments. These cages were approximately D1.8 × W1.2 × H2.4 m in size. Procedures were the same as for the outdoor experiments: food was placed in bottles with the trap lid, flies were released for 24–48 h before food bottles were collected, frozen and the number of flies in each bottle was determined. Indoor microcosms were on a 12-h light-dark cycle, and the temperature was kept at roughly 21°C.

### Live collection trap

3D-printed trap components were designed in the OpenSCAD scripting design language and printed in polylactic acid on a LulzBot Taz 6 printer (Fargo Additive Manufacturing Equipment). Designs are available at https://github.com/stenglein-lab/3D_parts/tree/master/fly_trap. For our initial trap testing, we used threadless fly bottles (VWR 75,813–140) and attached the trap lid to the bottle using parafilm. Subsequent experiments involving volunteer collectors used 4 oz jars (Uline, S-9934) and the 3D printed trap containing cornmeal-based food as described above. Before pouring the food into bottles, we added isoamyl acetate at a 1:100 ratio to food. Fifty mL of food was poured into bottles and left to cool overnight. Bottles were stored at 4°C until use. Each bottle was sealed with a cotton plug and parafilm. Traps were shipped to volunteers along with the 3D printed trap lid and parafilm. Volunteers were instructed to place the trap lid on the bottle and place the trap in a location with fruit fly activity (one outdoor location and one indoor location). Written instructions to volunteers are provided in supplemental texts 1 and 2. After ~1 week volunteers were instructed to seal the bottle with the provided parafilm and to ship it back to our lab (Figure 4a).

### Reusable trap

We created kits containing materials for trapping and returning flies. Kits included a clear plastic jar (Uline, S-9934), a 3D printed trap lid, a 3D printed barrier with organdie mesh (Oriole Textile, 2060), Fleischmann’s Instant Dry Yeast, a screw-top 2 mL tube and a pre-paid shipping return envelope. Volunteers were instructed to place 1–2 slices of banana in the bottom of the jar with a small pinch of yeast. The barrier was placed over the banana/yeast ensuring that flies remained separated from bait. Volunteers were instructed to replace the banana/yeast every 3–5 d depending on the environmental conditions (hot/humid environments were told to replace the banana/yeast more often) (Figure 4b). Flies were collected from traps that had been placed in a −20°C freezer for ≥30 min. Once dead, flies could be removed by tapping the jar upside down or with forceps and stored frozen at −20°C in 2 mL tubes. Tubes were returned in the provided prepaid shipping envelope.

### Data analyses

Data visualization and statistical analyses were performed in RStudio (v 2024.09.0 + 375) using the tidyverse package [26]. Analysis code is available at https://github.com/LKeene/fly_trap_design. A p-value of α = 0.05 or lower was used as our significance threshold. For attractiveness experiments, negative binomial models were generated using the MASS package, and comparisons were made using the emmeans package [27,28]. Pairwise comparisons were adjusted for multiple hypothesis testing using Tukey’s method. For Figure 3c, a pseudocount of one was added to all values to accommodate zero counts.

## Results

### Cornmeal-based fly food was most stable

We first set out to assess the stability of traps baited with different food substrates over 2 weeks. We tested cornmeal-, potato- and banana-based foods, all of which are commonly used to rear *D. melanogaster*. Each type was made plain or with synthetic banana flavouring (isoamyl acetate). Food bottles were placed in a controlled indoor environment or outside for 2 weeks. Bottles were visually assessed daily to identify signs of decay and microbial growth.

Banana-based food was the least stable with visible mould and bacterial growth occurring in a matter of days (Supplemental Figure S1). Potato-based food was more stable: after 2 weeks there was visible mould and bacterial growth but less than the banana-based food (Supplemental Figure S1). Cornmeal-based food was the most stable in indoor and outdoor environments (Supplemental Figure S1). The indoor cornmeal-based food looked nearly the same at the end of the 2 weeks as it did at the start. The presence of synthetic banana flavouring did not impact the stability of any of the foods. For all food types, there was considerable evaporation in the bottles that caused moisture to accumulate, particularly outdoors (Supplemental Figure S1).

We next sought to augment the attractiveness of cornmeal food baited traps. Although flies can be maintained on such food in the lab, it was unclear whether these foods would be sufficiently attractive. We expected that there would be competition from other food sources, particularly in outdoor environments, where organic volatiles known to attract *D. melanogaster* are emitted from rotting plant debris [29,30]. *D. melanogaster* are attracted to fermenting banana catalysed by the addition of yeast [31]. However, this banana and yeast mixture decays over the course of several days, creating a sticky substance that makes it difficult to retrieve flies. We therefore employed a choice assay with our wild derived laboratory line of *D. melanogaster* (FoCo-17) where the flies were exposed to plain cornmeal-based food with or without homemade banana extract or to banana/yeast bait [25].

**Figure 1.**
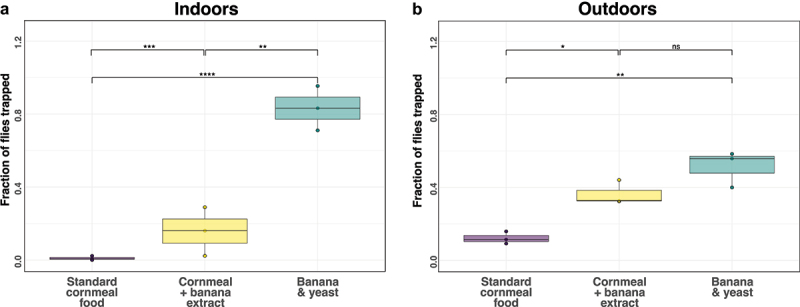
*D. melanogaster* were more attracted to banana-based baits. Fly bottles were either filled with plain cornmeal-based food, cornmeal-based food with homemade banana extract or banana and yeast. A 3D printed fly trap lid was used to seal the bottles. FoCo-17 flies were released in controlled environments either inside (*n* = 143, *n* = 197, *n* = 173) or outside (*n* = 173, *n* = 61, *n* = 220) and left for 24–48 h for a total of three independent experiments. Bottles were collected and the proportion of flies in each bottle was tabulated. Significance values from a negative binomial model are: *p* < 0.0001 ‘****’, 0.0001 < *p* > 0.001 ‘***’, 0.001 < *p* > 0.01 ‘**’, 0.01 < *p* > 0.05 ‘*’ and *p* > 0.05 ‘ns’.

We released flies into mesh enclosures in controlled indoor or outdoor environments containing traps baited with different food types. We left the flies for 24–48 h and recorded the number of flies in each bottle. Flies preferred food with a banana component (Figure 1). Banana and yeast food was more attractive than either plain cornmeal (p = 1.7×10^−10^) or cornmeal food supplemented with homemade banana extract (p = 5.4×10^−3^). This preference was less pronounced in outdoor conditions (p = 8.8×10^−3^ & p = 0.83, Figure 1). This shows that banana and yeast were an effective fly attractant, as expected. However, a banana-based additive also increased attractiveness.

### Banana extract was more attractive to *D. melanogaster* than marula tree derivatives

The fruit of the marula tree has been proposed to be the ancestral host of *D. melanogaster* [23]. We therefore tested whether ethyl isovalerate, a volatile ester emitted from marula fruit, would be better at attracting flies than banana-based compounds. Southern African *D. melanogaster* populations have polymorphisms in the Or22a odorant receptor that increase sensitivity to this compound, but this has not yet been tested using North American populations [23]. Since flies were highly attracted to banana, we also tested the use of synthetic banana flavouring (isoamyl acetate) as an inexpensive and readily available chemical attractant. Isoamyl acetate is also a prominent ester in marula fruit [23,32].

We also wanted to test whether the effectiveness of these additives was impacted by the food base. Fly bottles with cornmeal or potato food and the various attractants were placed in indoor or outdoor enclosures. Flies from the wild-derived FoCo-17 population were released for 24–48 h and the number of flies in each trap was recorded.

**Figure 2.**
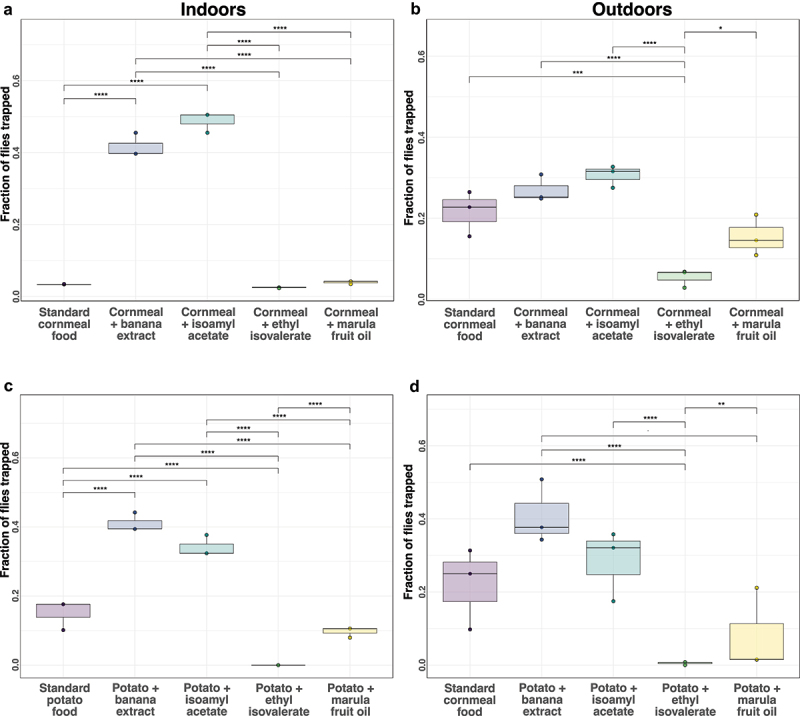
*D. melanogaster* prefer food with a banana-based attractant over food with derivatives of the marula fruit. The relative attractiveness of cornmeal-based (a & b) or potato-based (c & d) fly food with various baits were assessed in competition experiments. Experiments were conducted indoors (a,c) and outdoors (b,d). FoCo-17 flies were released for 24–48 h in enclosures containing baited fly traps. The number of flies in each replicate was: (a) indoor *n* = 121, 121, 88; (b) outdoor *n* = 211, 206, 378 and (c) indoor *n* = 142, 142, 138; (d) outdoor *n* = 134, 246, 260. Experiments were performed in triplicate. Significance values determined from a negative binomial model: *p* < 0.0001 ‘****’, 0.0001 < *p* > 0.001 ‘***’, 0.001 < *p* > 0.01 ‘**’, 0.01 < *p* > 0.05 ‘*’ and *p* > 0.05 ‘ns’.

Across all replicates in both environments, regardless of food type, banana-based odorants were the most attractive (Figure 2). Surprisingly, there were very few flies in the ethyl isovalerate or marula fruit oil bottles (Figure 2). There was a decrease in the relative attractiveness of banana extracts outdoors (Figure 2b,d). Standard cornmeal-based food with either homemade or store-bought banana extract produced statistically indistinguishable results (Figure 2). Due to the ease of use and chemical consistency, we opted to use synthetic banana flavouring (isoamyl acetate) for subsequent experiments.

The lack of attractiveness of ethyl isovalerate led us to test whether we were using an appropriate concentration. We performed an experiment using five ethyl isovalerate concentrations: 0.45%, 0.9%, 1.8% (~the natural concentration in marula fruit [32]), 3.6% and 7.2%. Plain cornmeal bottles and bottles with cornmeal plus isoamyl acetate at 1.5%, the natural concentration found in marula fruit [32], served as controls. As with the previous experiments, FoCo-17 flies were released in our indoor mesocosm for 24–48 h and then counted to determine the proportion of flies in each bottle.

**Figure 3.**
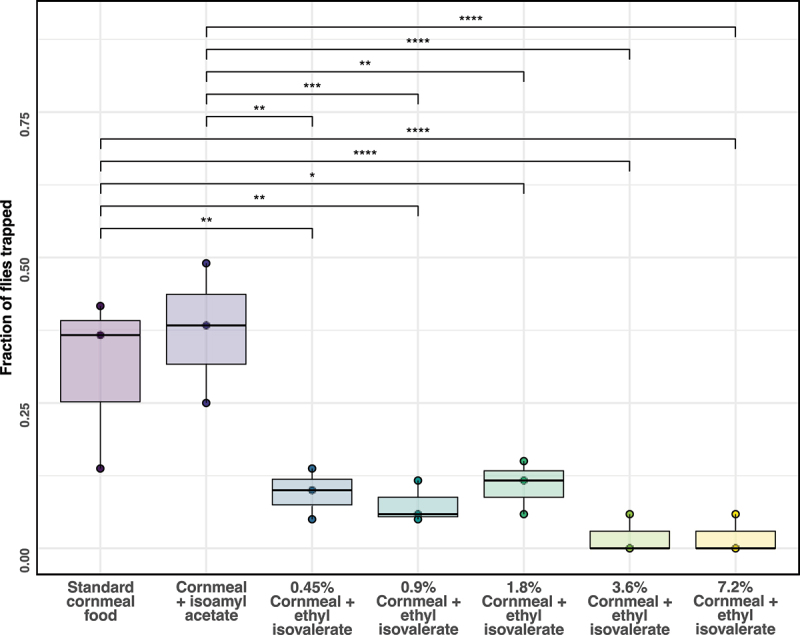
An outbred population of *D. melanogaster* from Colorado was not attracted to ethyl isovalerate. Flies from the FoCo-17 wild-derived outbred population of *D. melanogaster* were released (*n* = 51, 60, 60) in an indoor microcosm cage for 24–48 h. Cornmeal-based food with the following additives were placed in each cage: plain food, 1.5% synthetic banana extract and food supplemented with 0.45%, 0.9%, 1.8%, 3.6% or 7.2% ethyl isovalerate. After 24–48 hours the number of flies were counted. The experiment was performed in triplicate. Significance values determined from pairwise comparisons of a negative binomial model are: *p* < 0.0001 ‘****’, 0.0001 < *p* > 0.001 ‘***’, 0.001 < *p* > 0.01 ‘**’, 0.01 < *p* > 0.05 ‘*’ and *p* > 0.05 ‘ns’.

Flies were most attracted to plain food or food containing synthetic banana extract (Figure 3). The ethyl isovalerate bottles in aggregate attracted on average 31.8% of the flies with 1.8% ethyl isovalerate bottle attracting 10.5% of the flies. The 3.6% and 7.2% ethyl isovalerate bottles attracted the fewest flies. In contrast, synthetic banana flavouring attracted on average 37.4% of the flies and plain food 30.6% of the flies (Figure 3). Food with banana extract was not significantly more attractive than plain food. It is possible that the plain and banana extract food bottles were not placed far enough apart due to the limited space in the mesocosm. Overall, the results of these experiments confirmed our previous findings that *D. melanogaster* were more attracted to isoamyl acetate than ethyl isovalerate.

### A kit for capture of wild *D. melanogaster*

**Figure 4.**
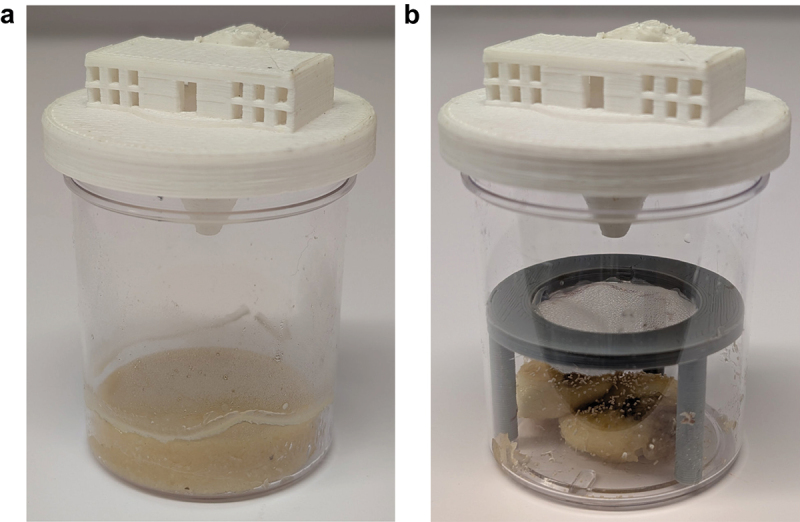
A 3D printed fly trap kit to collect wild *D. melanogaster*. Fly trap designs for the collection of (a) live flies, or (b) for repeated fly collection over time. Trap components are 3D printed. The version tested in this paper, designed to look like the White House, is depicted (the “flyte house”). Cornmeal-based food with isoamyl acetate can be used as a stable food that facilitates egg laying (a) or banana and yeast can be used as bait without the risk of flies becoming trapped in the decomposing fruit (b).

As a pilot test of the ability of these traps to be used by non-specialists, we sent traps with cornmeal-based food with isoamyl acetate to three volunteers in New York state (*N* = 1) and California (*N* = 2; Figure 4a). Volunteers were instructed to place the traps in an outdoor or indoor location with fruit fly activity for roughly a week (Supplemental Text 1). They were told to watch for trapped flies and signs of larval development. Once the first instar larva could be seen, the volunteers were instructed to seal the bottle with parafilm and ship it back to our lab. We received bottles from New York and California and were able to generate colonies that persist 3 years later.

We next tested a modified trap that simplified fly capture and trap reuse. This trap included a 3D printed ‘table’ covered in fine mesh to separate flies from the bait (Figure 4b). This trap design takes advantage of the attractiveness of banana/yeast bait while preventing flies from getting entrapped.

These traps proved to be an effective means to capture flies. Three additional volunteers from the US states of Maine, Ohio and Pennsylvania were instructed to add one to two slices of banana and a small pinch of yeast to the bottom of the jar, place the ‘table’ over the banana and close the jar with the trap lid (Supplemental Text 2). They then placed the trap in an area with fruit fly activity and monitored the trap for flies and rotting of the bait. Volunteers periodically froze the trap to kill the flies and moved them to the 2 mL tube. The tube was stored in the freezer and another round of collection began with fresh bait. At the end of the collection period (1–3 months) they shipped the 2 mL tubes back to our lab. Once at our lab, the fly species was determined using morphological characteristics and stored in a −80°C freezer until further analysis. The 32 flies collected from Pennsylvania and the 32 flies collected from Maine were *D. melanogaster*. Of the 17 flies collected from Ohio, only two were confirmed to be *D. melanogaster* using qRT-PCR targeting *D. melanogaster* RpL32 mRNA [33].

## Discussion

Here, we describe the design and effectiveness of two *D. melanogaster* fly trap designs. The first trap is characterized by a stable cornmeal and agar-based food supplemented with propionic acid to inhibit bacterial growth. Eggs laid by adult flies remained viable in this food even during shipment. This made it possible to generate new laboratory colonies from various locations after collection by non-specialists. We have maintained two stocks of *D. melanogaster* generated from flies collected in this manner in 2021.

The resulting populations could be used for a variety of experimental and observational purposes. However, a caveat is that flies may continue to mate following entrapment. This could produce shifts in allele frequencies in offspring, since sperm from the most recently mated male are more likely to fertilize eggs [34]. These traps are also likely not as suitable for the collection of large numbers of flies compared to, for instance, large, baited buckets [19].

The second trap was designed to facilitate simple sample collection and trap reuse. This makes it useful for crowdsourced sample collection and longitudinal collection. In a small field trial, kits were sent to nine volunteers with no previous *Drosophila* collecting experience. Volunteers were able to setup the kits and three locations successfully collected flies that were received by our laboratory.

We tested the attractiveness of marula fruit oil and ethyl isovalerate as *D. melanogaster* attractants because it had been proposed that ancestral populations of these flies evolved to specialize on marula fruit [23]. However, we found that banana-based additives were more attractive, at least to our wild-derived flies from Colorado (Figures 2 & 3). Although *D. melanogaster* are considered to be generalists, many species within the *melanogaster* subgroup specialize on a single fruit with populations of the same species showing different specializations [23,35,36]. It is possible that the population of *D. melanogaster* we tested have either lost sensitivity to ethyl isovalerate or gained sensitivity to volatiles released by different rotting fruits [23,30]. This could explain the observed unattractiveness of this compound. There is considerable variation in the odorant receptors that detect esters emitted by yeast and fermenting fruit [37]. Our analyses did not consider the possibility that some baits might be repellent to flies instead of just not attractive [38,39].

These traps could be modified or improved in a variety of ways. These traps could be combined with various baits targeting specific *Drosophila* species [19]. For instance, traps could be baited for the collection of agricultural pests like *D. suzukii* or flies in the Tephritidae family using alternative attractants like vinegar, methyl eugenol, isoamyl alcohol or cis-3-hexenyl acetate [20,21,40–42]. We did not exhaustively test different baits for *D. melanogaster*, and alternatives, including cantaloupe, may prove more attractive. Our 3D-printed trap lid was designed using the OpenSCAD 3D modelling software (https://openscad.org/). It is straightforward to use this software to modify the lid design, for instance to accommodate different size bottles or jars. Future studies will be necessary to evaluate the performance of these traps in different habitats or environmental conditions.

The use of community engagement to further scientific research has become increasingly popular [43–45]. The traps are designed for use by untrained volunteers and to be baited with readily available materials. *D. melanogaster* are attracted to a variety of other fruits if banana is unavailable [46]. Overall, our designs add another tool for the collection of wild flies by researchers and non-specialists alike to obtain flies from diverse locations for minimal cost and effort.

## Supplementary Material

Fly Trap Instructions.docx

SuppFig1.eps

## Data Availability

The raw data that support the findings of this study and the scripts that were used to generate the figures are available on GitHub at https://github.com/LKeene/fly_trap_design.
