# Supplementary material for: Experimental assessment of 3D-printed traps and chemical attractants for the collection of wild Drosophila melanogaster
Source: Fly (Austin). 2025 May 14;19(1):2502184. doi: 10.1080/19336934.2025.2502184 (PMC12087654; doi:10.1080/19336934.2025.2502184)
Supplement: Fly Trap Instructions.docx [file KFLY_A_2502184_SM4498.docx]

# of samples

**
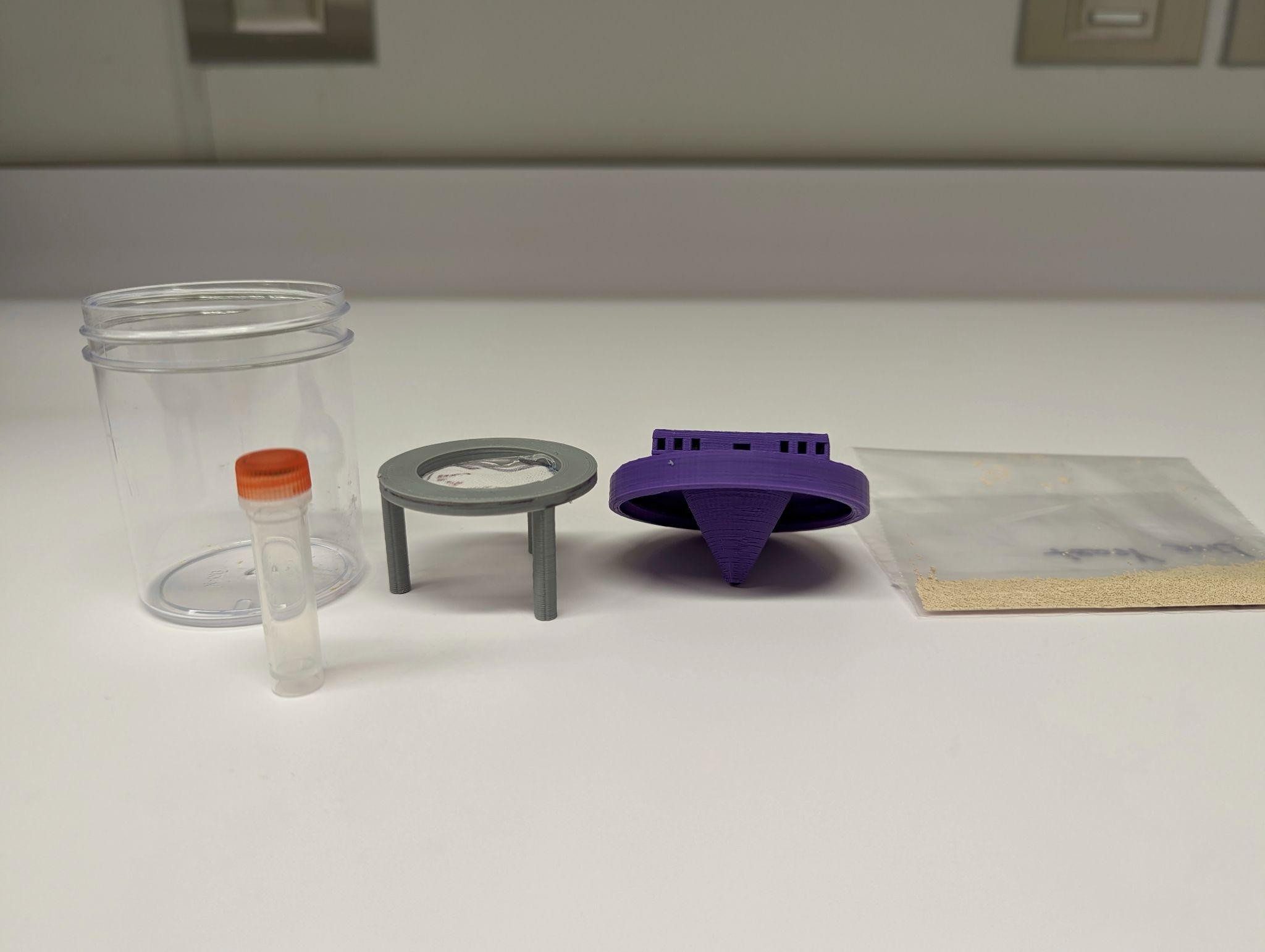
Package Contents :**

- 1 Jar with original lid
- 1 3D printed “Flyte House” fly trap lid
- 1 3D printed mesh table
- 1 Bag of Yeast
- 3 2 mL tubes
- 1 return envelope and a return label

# What you provide

- Banana [if food not already provided in trap]

1. If you have been provided a trap with food in it, please proceed to step 4 and do not place the mesh table into the trap.
2. Add a small slice of banana (the thickness of a few quarters stacked on top of each other) to the bottom of the empty jar and sprinkle a very small pinch of yeast on top of the banana.
3. Place the “table” into the jar (it will be snug). If the banana touches the mesh on the “table” you have put too much banana in, please take some out before continuing.
4.
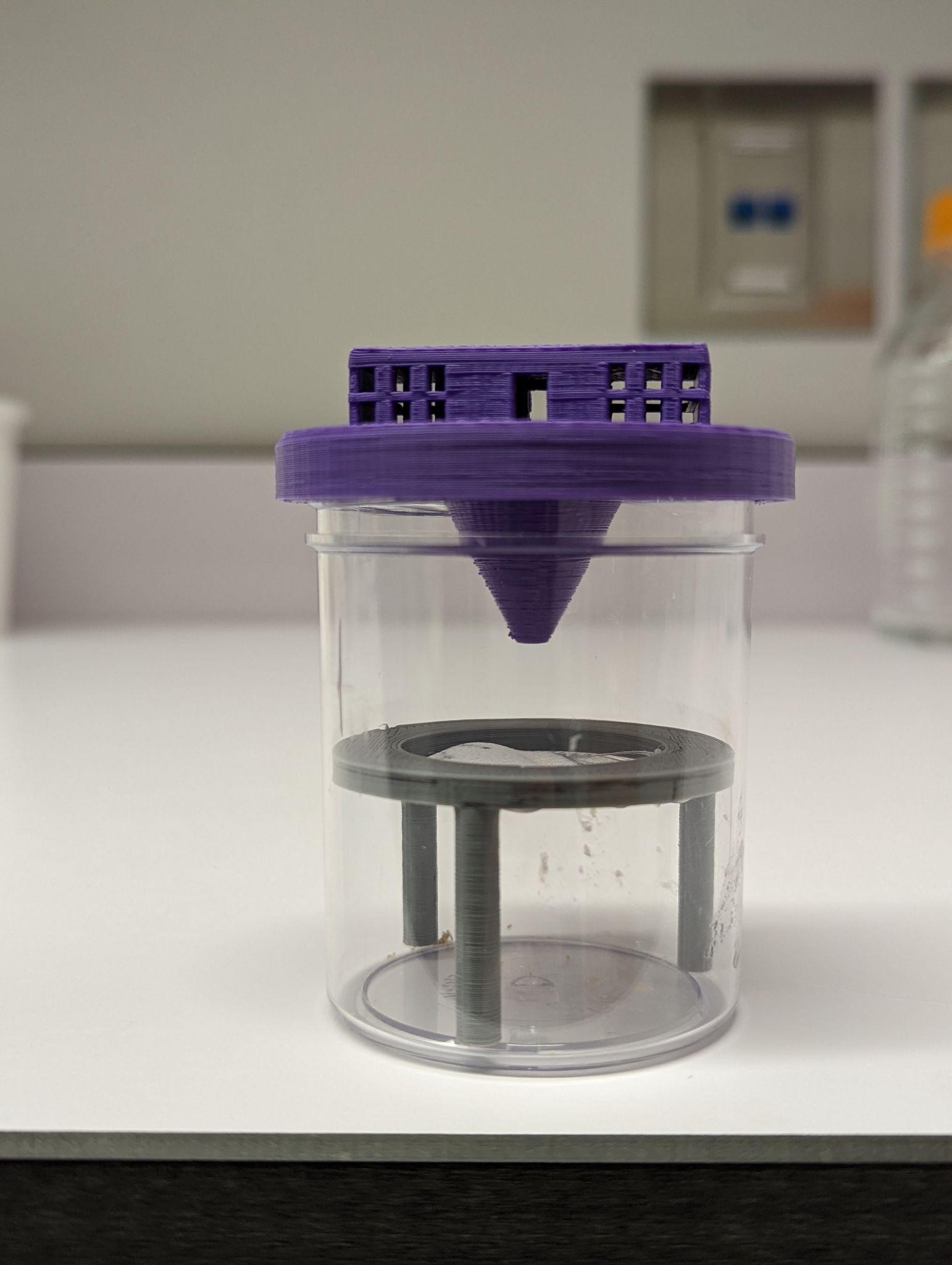
Screw on the fly trap lid (flyte house) to the jar
5. Place the jar in a location where you expect to find fruit flies. Some good locations include in the kitchen next to fruit or outside next to a compost pile, fruit tree, or garbage can.

*If placing the jar outside try to put it somewhere it won’t be disturbed by other people or animals.

1. Keep an eye on the trap for up to 2 weeks but if your trap collects 12 or more flies before then you may stop collecting early. Below is an image of the type of fruit fly we are looking for. Other insects may get in the traps. That is okay, we will identify everything that gets to us. Remove the banana and wash the trap before using it again.


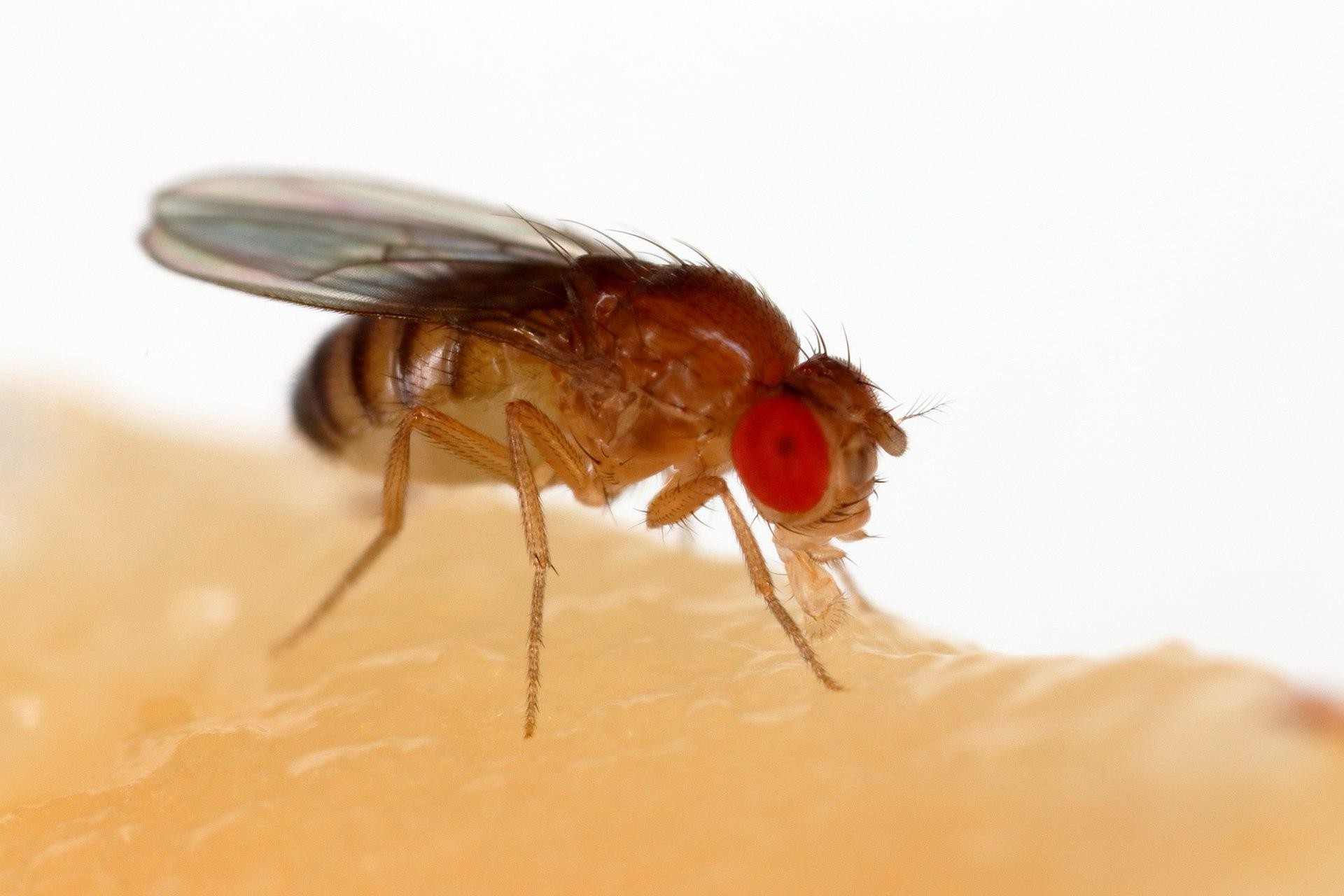


https://commons.wikimedia.org/wiki/File:Drosophila_melanogaster_Proboscis.jpg

1. Follow the instructions in the next section to return flies to us.
2. Once you are ready to end the experiment you will need to transfer flies to the sample tube from your kit to send back to us.
3. Take the jar and place it in your freezer for **1 hour** (If you do less than 1 hour, the flies may wake up while you try to move them).
4. Remove the jar from the freezer and gently tap it on a counter so the flies fall to the bottom of the trap (i.e. on top of the mesh table).
5. Remove the lid and gently tap the flies onto a clean paper towel and allow them to dry for **1 day**. Allowing the flies to dry out before shipping will keep them from sticking together in the tube.
6. Once the flies are dried, gently transfer them into one of the small sample tubes provided in your kit. Please label the tube with your name, the date the flies were collected, and the location.
7. Place the tube into the prepaid envelope and tape the return label you received in your kit and go to the nearest FedEx to mail it like any other letter. If needed, you can leave the flies in the freezer until you are able to mail it.

# Instructions for shipping traps to our lab: return of traps for establishing colonies

1. If your trap has collected female flies, it is likely that they will have laid eggs into the provided food. You will need to remove the fly trap lid and put on the original jar lid.
2. Place the trap into your freezer for **1-1.5 minutes** (no longer or the flies will die). During this time get the original lids ready for the switch. You will need to be quick. This causes the flies to go to sleep but not for long!
3. Open the freezer, gently tap the jar on a counter so the flies fall to the bottom and quicly switch the lid from the trap lid back to the original lid. If flies escape that is OK - you may want to do this outside in case any do escape.
4. Repackage the jars using the same box that was sent to you, there should be a return label in the package that you can attach. Then ship it out to us here in Fort Collins.
